# Low grain weight, a new allele of BRITTLE CULM12, affects grain size through regulating *GW7* expression in rice

**DOI:** 10.3389/fpls.2022.997624

**Published:** 2022-09-13

**Authors:** Yafeng Ye, Shuoxun Wang, Yan Ren, Huijie Yang, Junyao Guo, Hongrui Jiang, Xiaotong Zhu, Wenhao Li, Liangzhi Tao, Yue Zhan, Yuejin Wu, Xiangdong Fu, Kun Wu, Binmei Liu

**Affiliations:** ^1^Key Laboratory of High Magnetic Field and Ion Beam Physical Biology, Hefei Institutes of Physical Science, Chinese Academy of Sciences, Hefei, China; ^2^Anhui Province Key Laboratory of Environmental Toxicology and Pollution Control Technology, Hefei Institutes of Physical Science, Chinese Academy of Sciences, Hefei, China; ^3^State Key Laboratory of Plant Cell and Chromosome Engineering, Institute of Genetics and Developmental Biology, Chinese Academy of Sciences, Beijing, China

**Keywords:** grain size, rice, grain yield, LGW, *GW7*, gene expression

## Abstract

Grain weight is a major determinant in rice yield, which is tightly associated with grain size. However, the underlying molecular mechanisms that control this trait remain unclear. Here, we report a rice (*Oryza sativa*) mutant, low grain weight (*lgw*), which shows that reduced grain length is caused by decreased cell elongation and proliferation. Map-based cloning revealed that all mutant phenotypes resulted from a nine-base pair (bp) deletion in *LGW*, which encodes the kinesin-like protein BRITTLE CULM12 (BC12). Protein sequence alignment analysis revealed that the mutation site was located at the nuclear localization signal (NLS) of LGW/BC12, resulting in the lgw protein not being located in the nucleus. LGW is preferentially expressed in both culms and roots, as well as in the early developing panicles. Overexpression of *LGW* increased the grain length, indicating that LGW is a positive regulator for regulating grain length. In addition, LGW/BC12 is directly bound to the promoter of *GW7* and activates its expression. Elevating the *GW7* expression levels in *lgw* plants rescued the small grain size phenotype. We conclude that LGW regulates grain development by directly binding to the *GW7* promoter and activating its expression. Our findings revealed that LGW plays an important role in regulating grain size, and manipulation of this gene provides a new strategy for regulating grain weight in rice.

## Introduction

Rice is a staple food for more than half of the world’s population. Improving rice yield is crucial to meet the rapid growth of the global population. Rice yield is mainly determined by three major parameters: panicle number per plant, grain number per panicle, and grain weight. Grain weight is determined by the grain size and degree of grain filling. The cell number and cell size of the spikelet hull directly determine rice grain size (Li et al., 2018, 2019). Several quantitative trait loci (QTLs) and several genes that regulate grain size have been identified in recent studies (Wang et al., 2012, 2015; Duan et al., 2014, 2017; Che et al., 2015; Liu et al., 2015, 2017, 2018; Huang et al., 2017; Hao et al., 2021). The main regulatory pathways of grain size include the G protein, mitogen-activated protein kinase (MAPK), ubiquitin–proteasome, and phytohormonal signaling pathways and transcriptional regulatory factors (Xu et al., 2019).

In rice, all G-protein subunits participate in grain size regulation. Mutation of *RGA1*, which encodes a Gα subunit in rice, results in a severely dwarf plant and produces small grains (Sun et al., 2018). Reduced expression of RGB1, which encodes the Gβ subunit, leads to short panicles and small grains in rice (Utsunomiya et al., 2011). Therefore, RGA1 and RGB1 are positive regulators of rice grain size. However, genes encoding Gγs subunits play diverse roles in regulating grain size. Overexpression of both *RGG1* and *RGG2* leads to small grains, suggesting that RGG1 and RGG2 are negative regulators of rice grain size (Miao et al., 2019; Tao et al., 2020). GS3 and DEP1 are the two major QTLs that play antagonistic roles in grain size (Sun et al., 2018). Different mutations in GS3 result in different grain sizes. Loss of *GS3* function results in a long-grain phenotype (Fan et al., 2006). However, the *GS3-4* allele, which encodes a truncated protein retaining the G-protein γ-like (GGL) domain in the N-terminal tail, has a dominant negative effect on grain size. Overexpression of the wild-type *GS3* and *GS3-4* alleles results in small grains (Mao et al., 2010). Therefore, GS3 acts as a negative regulator of grain size. In rice, DEP1 plays multiple roles in plant development, including regulation of grain size, panicle development, and responses to nitrogen (Huang et al., 2009; Sun et al., 2014; Liu et al., 2018). GGC2, as the closest homolog of DEP1, either individually or in combination, regulates grain size when complexed with Gβ (Sun et al., 2018). In rice, the OsMKKK10-OsMKK4-OsMAPK6 cascade has been reported to regulate grain size, OsMKKK10 phosphorylates and activates OsMKK4 and OsMAPK6, and the activity of OsMAPK6 is positively associated with grain size (Xu et al., 2018a,b; Guo et al., 2020). Rice OsMKP1, which encodes a MAPK phosphatase, plays an antagonistic role with OsMKK4 regulating OsMAPK6 activity and grain size (Xu et al., 2018b). Therefore, accurate regulation of OsMPK6 activity through reversible phosphorylation is key to rice grain size regulation. *GW2*, which encodes a RING-type E3 ubiquitin ligase, negatively regulates grain width (Song et al., 2007). GW2 ubiquitinates WG1 and modulates its stability, thereby relieving the inhibition of OsbZIP47 transcriptional activity and promoting downstream genes transcription to decrease the grain width (Hao et al., 2021). The deubiquitinating enzyme OsOTUB1/WIDE AND THICK GRAIN 1 (WTG1) controls grain size and shape mainly by influencing cell expansion (Huang et al., 2017). Some factors of the brassinosteroid (BR) signaling pathway, such as OsBRI1, OsBAK1, OsBZR1, qSW5/GW5, and qGL3/GL3.1, have been found to regulate grain size (Duan et al., 2017; Liu et al., 2017). Transcriptional regulatory factors, such as SQUAMOSA promoter-binding protein-like family transcription factors, SPL13 and SPL16, are positive regulators of grain length by promoting cell expansion or/and cell proliferation in the spikelet hull by regulating *SRS5* and *GW7* expression, respectively (Wang et al., 2015; Si et al., 2016). GW7 encodes a homolog of Arabidopsis LONGIFOLIA protein, which regulates longitudinal polar cell elongation (Wang et al., 2015).

Kinesins are ATP-driven microtubule-based motor proteins that transport vesicles containing cytoplasmic cargo to specific destinations in higher eukaryotes (Schuyler et al., 2003). Kinesins are divided into 14 subfamilies according to their conserved motor domains. The kinesin-4 subfamily shares a highly conserved ATPase domain at the N-terminal, a globular domain at the C-terminal, and a coiled-coil domain in the middle region (Mazumdar and Misteli, 2005). Most members of the kinesin-4 subfamily in animals have putative nuclear localization signals (NLSs) and perform nuclear functions (Murphy and Karpen, 1995; Walczak et al., 1998; Levesque and Compton, 2001; Canman et al., 2006). In plants, the *Arabidopsis* kinesin-4 protein, FAR1, is only found in the cytoplasm and is involved in cellulose microfibril order and cell elongation (Kong et al., 2015). However, the rice BRITTLE CULM 12 (BC12), a homolog of FRA1, has an NLS and is located in both the cytoplasm and the nucleus (Zhang et al., 2010). Mutation of *BC12* results in brittle cum and dwarfism phenotypes by affecting cellulose microfibril deposition and regulating the GA biosynthesis pathway, respectively (Zhang et al., 2010; Li et al., 2011). However, the mutation also leads to small grain size, and the molecular mechanism underlying the BC12-mediated regulation of grain size is still unclear.

In this study, we reported a novel mutant allele of *BC12*, which showed dwarfism and small grain without brittle culm phenotype that we have called *lgw*. Our data show that the mutation site was located at the NLS of LGW/BC12, resulting in the lgw protein not being located in the nucleus. Further research has shown that LGW/BC12 was directly bound to the promoter of *GW7* and regulated its expression. *GW7* overexpression in *lgw* plants rescued the small grain size phenotype. Therefore, LGW regulates grain size by directly binding to the *GW7* promoter and regulating its expression.

## Materials and methods

### Plant materials and growth conditions

The *lgw* mutant was isolated from *japonica* cv. Wuyunjing7 (WYJ7) using a heavy ion beam treatment. The *lgw* mutant was crossed with *indica* cv. 9,311 to generate a mapping population. All rice plants used in this study were grown in experimental fields at the Hefei Institute of Physical Science, the Chinese Academy of Sciences (Hefei, China), and Sanya (Hainan Province, China) in the natural growing season.

### Scanning electron microscopy

Naturally dried mature grains were sputter-coated with gold and observed under a scanning electron microscope (SEM; S570; Hitachi). The cell number, cell length, grain length, and width were measured using the ImageJ software.

### Map-based cloning

The *lgw* locus was mapped and cloned using 2,217 mutant plants from the F_2_ population as described above. Simple sequence repeat (SSR) markers with polymorphism distributed uniformly throughout the whole rice genome were used for the *lgw* locus rough mapping. For fine mapping, we designed insertion/deletion (Indel) markers in the rough mapping region. Candidate genes in the 114-kb region were amplified from *lgw* and WT plants using KOD DNA polymerase (TOYOBO) and sequenced using an Applied Biosystems 3730 sequencer. For complementation of the *lgw* mutant, a 5.6-kb genomic fragment was inserted into the pCAMBIA1300 vector to generate the construct *pLGW:LGW* (*pLGWF*). *pLGWF* was introduced into the *lgw* mutant using the *Agrobacterium*-mediated transformation protocol as described previously (Ye et al., 2018).

### RNA extraction and quantitative real-time polymerase chain reaction

RNA from various rice tissues was extracted using TRIzol reagent (Invitrogen), as described previously (Ye et al., 2015). cDNA was synthesized using a reverse transcription kit (TransGen). qRT-PCR was carried out on a Roche LightCycler 480 with SYBR Green Supermix (TransGene) according to the manufacturer’s instructions. The primers used for qRT-PCR are shown in Supplementary material. All assays were repeated three times, and *Actin1* was used as an internal control.

### Chromatin immunoprecipitation analysis

The 3–6 cm length panicles of *pActin:LGW-Flag* transgenic rice plants having approximately 3 g were harvested and immediately fixed in 1% formaldehyde for cross-linking under vacuum for 15 min at room temperature. Cross-linking was stopped by adding 0.125 M glycine for 5 min under vacuum. The cross-linked samples were rinsed at least two times with ddH_2_O. ChIP assays were performed as previously described (Ye et al., 2018). Chromatin samples were mixed with anti-Flag (Sigma, St Louis, MO, United States; F1804) antibodies for immunoprecipitation. The enrichment of DNA fragments was determined by a qRT-PCR analysis performed on three biological replicates. Relevant PCR primer sequences are listed in Supplementary material. The *Actin1* gene exon was used as a negative control.

### Subcellular localization analysis

To observe LGW and *lgw* subcellular localization, the green fluorescent protein (GFP) was fused to its C-terminus and inserted into the pCAMBIA2300 driving by cauliflower mosaic virus (CaMV) 35S promoter. The expressed LGW-GFP and lgw-GFP in rice protoplast and *Nicotiana benthamiana* leaves were observed using a confocal laser scanning microscope (Zeiss LSM700).

### Transactivation analysis in rice protoplasts

The *LGW* coding sequence was amplified and fused to the GAL4 binding domain (GAL4BD) to generate an effector. Empty GAL4BD was used as a negative control. The 2.7-kb upstream sequence from ATG of *GW7* was amplified and fused with the LUC protein to generate a reporter, and the corresponding vector of *BC11* was used as a negative control. A pTRL plasmid containing the Renilla LUC gene driven by the 35S promoter was used as an internal control. The transactivation assay was performed as described previously (Wang et al., 2015). The pTRL, effector, and reporter were simultaneously transformed into the rice protoplast system and kept in the dark overnight. LUC activity was determined as previously described (Liu et al., 2018).

### Yeast one-hybrid assay

The *LGW* encoding sequence was amplified and inserted into the *pB42AD* vector (Takara) to generate the effector. The 2.7-kb upstream sequence from the ATG of *GW7* was amplified and inserted into the pLacZi2μ vector to generate the reporter. Y1H assays were performed as previously described (Ye et al., 2018). The effector and the reporter were simultaneously transformed into yeast strain EGY48. The clones were grown on culture medium plates without tryptophan, uracil, and using β-D-galactopyranoside for colony coloration. Empty pLacZi and pB42AD were used as negative controls.

### Statistical analysis

We used SPSS software version 21.0 to analyze all data. The pairs of means were compared by Student’s *t*-test, while comparisons between multiple groups were performed using ANOVA followed by Duncan’s multiple range test.

## Results

### The low grain weight mutant exhibits reduced grain length by decreasing cell elongation of spikelet hull

**FIGURE 1:**
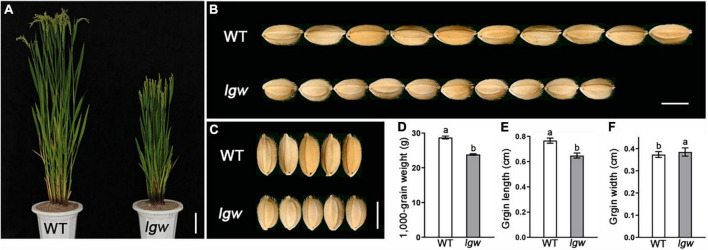
Comparison of the grain size between wild type (WT) and *lgw*. **(A)** Three-month-old plant of wild-type (WT) and *lgw* mutant. Bar = 10 cm. **(B,C)** Observation of grain length **(B)** and grain width **(C)** of WT and *lgw*. Bar = 0.5 cm. **(D)** 1,000-Grain weight. **(E)** Grain length. **(F)** Grain width. Error bars represent SE (*n* = 30). Different letters denote significant differences (*P* < 0.05) from Duncan’s multiple range test.

A low grain weight (*lgw*) mutant was screened and isolated from plants of the *japonica* cultivar Wuyunjing7 (WYJ7) mutagenized with a heavy ion beam. Compared with the wild type (WT), the *lgw* mutant plants had reduced plant height, owing to small panicles and shortened internode (Figure 1A and Supplementary Figure 1). In addition, the *lgw* mutant exhibited a small grain phenotype (Figures 1B,C). The 1,000-grain weight decreased by 20.62% compared to that of the WT grains (Figure 1D). Similarly, the length of *lgw* grains decreased by 15.44% compared with that of the WT grains (Figure 1E). However, the width of the lgw grains increased by 3.12% compared with that of the WT grains (Figure 1F).

**FIGURE 2:**
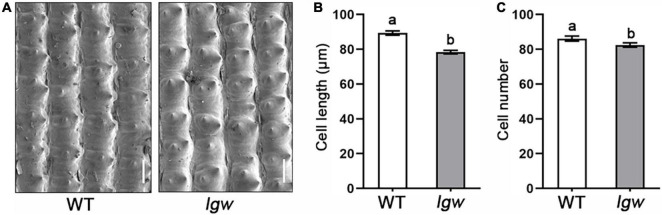
Histological and cytological observations of WT and *lgw*. **(A)** SEM observation of the grain husk of WT and *lgw* (Bar = 50 μm). **(B)** Cell length. **(C)** Cell number. Different letters denote significant differences (*P* < 0.05) from Duncan’s multiple range test. Error bars represent SE (*n* = 30).

The final cell number and the cell length in the spikelet hull determine the grain size. Therefore, we used SEM to observe the grain husks in the *lgw* mutant and show that, compared with the WT (Figure 2A), cell length in the spikelet hull decreased by approximately 11.36% (Figure 2B); however, the cell number of spikelet hulls decreased by approximately 4.35% (Figure 2C). These results suggest that the small grain size in the *lgw* mutant is mainly attributed to limited cell expansion and a slight decrease in the cell number of spikelet hulls.

### Isolation of low grain weight gene

**FIGURE 3:**
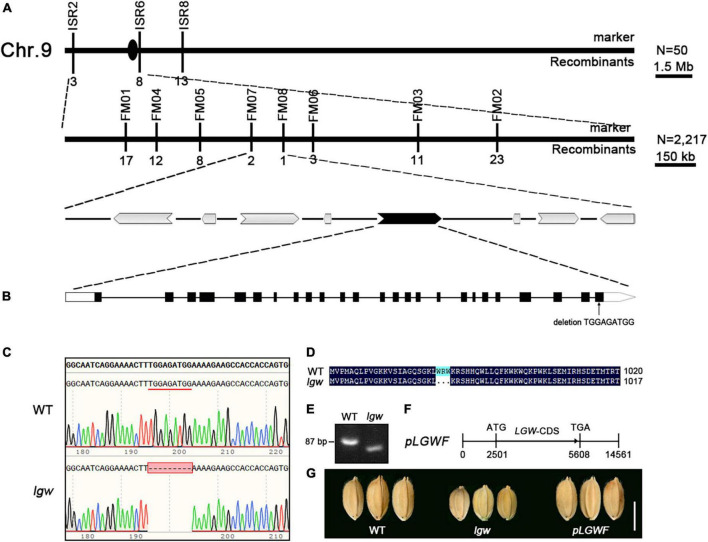
Map-based cloning of the *LGW* gene. **(A)** The *LGW* locus was mapped on chromosome 9 approximately 114 kb region containing 8 predicted ORFs. The numbers below the molecular markers represent the number of recombinants. **(B)** A fragment deletion of TGGAGATGG in LOC_Os09g02650. **(C)** DNA sequence alignments of WT and *lgw*. **(D)** Deduced LGW amino acid sequence alignments for WT and *lgw*. **(E)** The designed marker for confirming the genotypes of WT and *lgw*. **(F)** A construct for complementary assay. **(G)** Comparing the shape showed seeds recovered from complemented plants was consistent with that of WT. Bar = 0.5 cm.

To investigate the molecular basis of the *lgw* phenotypes, we first crossed the *lgw* mutant with its WT, all F_1_ individuals exhibited the WT phenotype. The F_2_ population contained 518 normal and 168 small grain size and dwarf plants [*x*^2^ (3:1) = 0.095 < *x*^2^0.05 = 3.84; *P* > 0.05], suggesting that the small grain size and dwarf phenotype of the *lgw* mutant is controlled by a single nuclear recessive gene. To map the *LGW* locus, we crossed the *lgw* mutant with 9311, a wild-type polymorphic *indica* cv. to generate a mapping population, and used a map-based cloning approach to isolate *LGW*. The *lgw* locus was located between molecular markers ISR2 and ISR6 on chromosome 9 (Figure 3) and then pinpointed within an approximate 114-kb region between Indels FM07 and FM08 (Figure 3A). The 114-kb region contained eight putative open-reading frames (ORFs) (Figure 3A). We then sequenced the eight ORFs and found a 9-bp deletion in LOC_Os09g02650 (Figures 3B,C). The deletion occurred in the last exon of the ORF, resulting in a three amino acid deletion in its protein sequence (Figure 3D). The mutated site was confirmed by amplifying the DNA fragment covering the deletion site (Figure 3E). To further confirm that the dwarf and small grain phenotype was caused by the mutation of LOC_Os09g02650, a 5.6-kb DNA fragment containing the 2.5-kb putative promoter and the coding sequence was cloned into the vector *pCAMBIA1300* to generate the complementation plasmid *pLGW:LGW* (named *pLGWF*) (Figure 3F), which was introduced into the *lgw* mutant plants by *Agrobacterium*-mediated transformation. As expected, positive transgenic *pLGWF* plants completely rescued the small grain size (Figure 3G) and dwarfism phenotypes (Supplementary Figure 2). Together, these results demonstrate that LOC_Os09g02650 is the *LGW* gene responsible for the mutant phenotypes described above.

### Low grain weight is ubiquitously expressed in many organs

**FIGURE 4:**
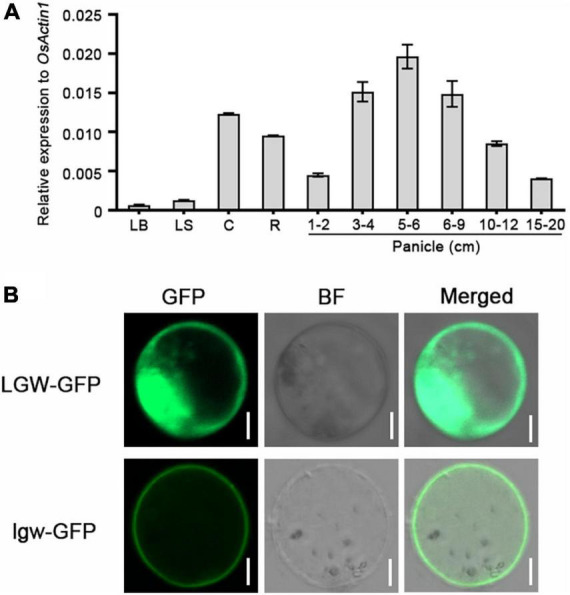
Expression pattern and subcellular localization of LGW. **(A)** The expression level of *LGW* in various rice organs. LB, leaf blade; LS, leaf sheath; C, culm; R, root; Panicle (1–2, 3–4, 5–6, 6–9, 10–12, 15–20 cm). The *Actin1* was used as an internal control. **(B)** The subcellular localization of LGW and lgw. LGW-GFP and lgw-GFP were expressed in rice protoplast, bars = 20 μm.

To analyze the expression pattern of *LGW*, we determined the *LGW* expression levels in various tissues of wild-type WYJ7 using quantitative real-time polymerase chain reaction (qRT-PCR). The results illustrated that *LGW* transcripts were detected in all organs but predominantly expressed in internodes, roots, and young panicles. The expression levels of *LGW* were relatively low in leaves and sheaths (Figure 4A). The ubiquitous expression of *LGW* was consistent with the phenotype of the *lgw* mutant, suggesting that *LGW* might play an important role in panicle and grain development.

### Low grain weight encodes a kinesin-4 protein BRITTLE CULM12

The full-length *LGW* CDS has 3,108 nucleotides that encode a protein of 1,305 amino acids with a predicted molecular mass of 117 kDa. The rice database annotates LGW as a kinesin-4 protein, named BC12 (Zhang et al., 2010). Phylogenetic analysis revealed that LGW was most similar to FAR1 (Supplementary Figure 3), which has been reported to be involved in cellulose microfibril deposition in *Arabidopsis*. Although BC12 is the closest homology to FRA1, differences were observed in the leucine zipper domain and NLS between BC12 and FRA1. As previously reported, the 17 amino acid sequence (amino acids 971–987) in BC12 is a functional NLS (Zhang et al., 2010). Protein alignment analysis revealed that the lgw mutation site (amino acids 983–985 deletion) was located in this NLS domain (Figure 3D). To determine whether the three amino acids in lgw affected its nuclear localization, we determined the subcellular localization of the WT form, LGW-GFP, and the mutated form, lgw-GFP. The results reveal that the fluorescent signals of LGW-GFP were targeted to the nucleus and the cytoplasm (Figure 4B and Supplementary Figure 4), which is consistent with the subcellular localization of GDD1/BC12 (Zhang et al., 2010; Li et al., 2011). However, the fluorescent signals of lgw-GFP were targeted to the cytoplasm rather than the nucleus (Figure 4B and Supplementary Figure 4). Mutation of BC12/GDD1 resulted in a brittle culm phenotype (Zhang et al., 2010; Li et al., 2011), but the *lgw* mutant only exhibits dwarf and small grains without the brittle culm phenotype (Supplementary Figure 5). These results suggest that the NLS of LGW is essential for plant height and grain development but is not required for secondary cell wall formation.

### Overexpression of low grain weight increases grain length and weight

**FIGURE 5:**
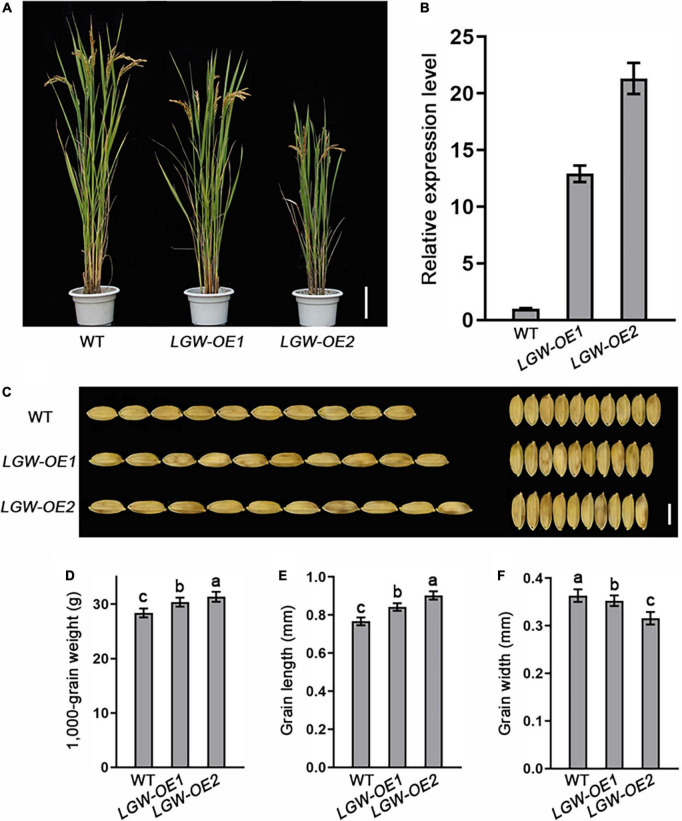
Overexpression of *LGW* increases grain length. **(A)** Morphology of mature LGW-OE1 and LGW-OE2 plants. Scale bar = 10 cm. **(B)** The relative expression level of *LGW* in WT, LGW-OE1, and LGW-OE2 lines. **(C)** Grain size, bar = 0.5 cm. **(D)** 1,000-Grain weight. **(E)** Grain length. **(F)** Grain width. Different letters denote significant differences (*P* < 0.05) from Duncan’s multiple range test.

To explore the biological function of *LGW* in controlling grain size and weight, we generated an LGW overexpression construct and transformed it into WYJ7. We selected two homozygous independent lines with significantly elevated expression levels of *LGW*, *LGW*-OE1, and *LGW*-OE2 (T3 generation) for further study (Figures 5A,B). Plant height decreased with increasing *LGW* expression levels (Figure 5A). The grain length (Figures 5C,D) and grain weight (Figure 5E) of overexpression lines increased significantly compared with that of the WT, and the grain width of the overexpression lines decreased significantly compared with that of the WT (Figures 5C,F). These were affected by the expression levels of *LGW*. These results suggest that LGW is a positive regulator of grain length and weight.

### Low grain weight regulates grain size by directly regulating *GW7* expression

**FIGURE 6:**
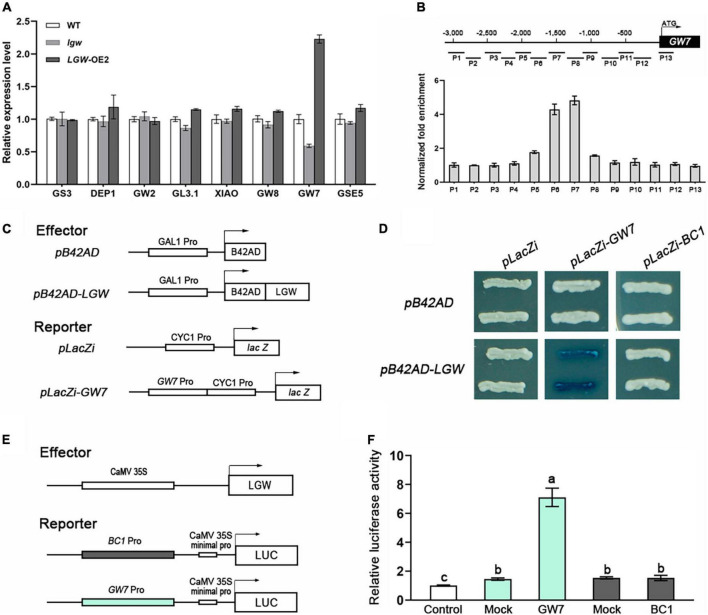
LGW directly regulates *GW7* expression. **(A)** Expression analysis of grain size regulators in WT, *lgw*, and LGW-OE2 plants. **(B)** ChIP assay. LGW-Flag mediated ChIP-qPCR enrichment (relative to Input) of CCA(N)nTGG-containing promoter fragments from *GW7*. Error bars, SE of three biological replicates. **(C)** Diagrams of the reporter and effector constructs used in **(D)**. **(D)** Yeast one-hybrid assays. LGW activates *LacZ* reporters driven by *GW7*. The empty pLacZi and pB42AD were used as a negative control. **(E)** Diagrams of the reporter and effector constructs used in **(F)**. **(F)** LGW activates transcription of the *GW7*. Mock, co-expressed with the reporter and an empty effector construct; control, co-expressed with an effector and an empty reporter construct (set to 1). Error bars, SE of three biological replicates. Different letters denote significant differences (*P* < 0.05) from Duncan’s multiple range test.

To investigate the molecular mechanism underlying *LGW* regulation of grain size, we first examined the expression levels of genes that have been previously reported to act as grain size regulators. qRT-PCR assay results reveal that the *GW7* expression was significantly downregulated in *lgw* plants and upregulated in *LGW*-OE2 plants (Figure 6A). There was no significant difference in the expression levels of the other genes in the WT, *lgw*, and *LGW*-OE2 plants (Figure 6A).

As previously reported, the BC12/GDD1 protein contains a Leu zipper motif, which was the conserved Leu residues in the bZIP transcription factors. bZIP proteins have been proposed to bind the element, CCA(N)_*n*_TGG (Li et al., 2011). Alignment analysis revealed that the upstream 3,000 bp sequence of the *GW7* promoter has seven candidate elements (Supplementary Table 1), indicating that LGW may directly bind to these elements to regulate *GW7* expression. To further verify whether LGW directly binds to these elements in the *GW7* promoter, we performed chromatin immunoprecipitation (ChIP) assays in WT and *LGW*-OE2 plants. ChIP assay results reveal that the two fragments (P6 and P7) containing CCA(N)_*n*_TGG elements were significantly enriched in *LGW*-OE2 plants (Figure 6B). We also used the yeast one-hybrid (Y1H) system to confirm the interaction between LGW and the *GW7* promoter (Figure 6C). Y1H assay results reveal that LGW activated *LacZ* reporter gene expression under the control of the *GW7* promoter. However, empty pB42AD did not activate *LacZ* expression (Figure 6D).

To examine the effect of LGW on the transcriptional regulation of *GW7* expression, dual-luciferase reporter (DLR) assays were performed in rice protoplasts. DLR assay results show that the luciferase activity in protoplasts co-expressing an effector carrying LGW and a reporter containing the *GW7* promoter to drive luciferase increased sevenfold, compared to the negative control (Figures 6E,F). This result indicates that LGW functions as a transcriptional activator that directly regulates *GW7* expression.

**FIGURE 7:**
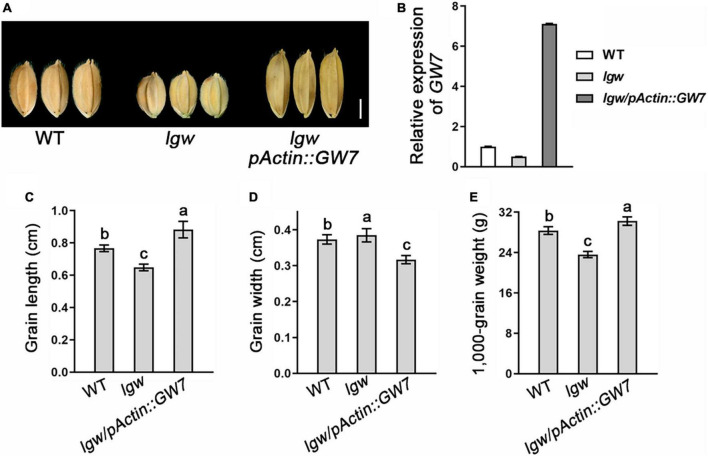
Elevating *GW7* expression level in *lgw* plants can rescue the small grain size phenotype. **(A)** Grain shape observation of WT, *lgw*, and *lgw/pActin:GW7*. Bar = 0.3 cm. **(B)** Expression level of *GW7* in WT, *lgw*, and *lgw/pActin:GW7*. **(C)** Grain length. **(D)** Grain length. **(E)** 1,000-Grain weight. Different letters denote significant differences (*P* < 0.05) from Duncan’s multiple range test.

Considering that LGW directly binds to the promoter of *GW7* and regulates its expression, we investigated whether elevating *GW7* expression levels in *lgw* plants can rescue the small grain size phenotype. Therefore, we generated the overexpression construct of GW7 (*pActin:GW7*) and transferred it into the *lgw* plants. Consistent with this notion, the overexpression of *GW7* in *lgw* mutants rescued the grain length and the grain weight phenotypes of *lgw*, which indicates that the small grain size and *lgw* in the *lgw* mutant rely on *GW7* expression levels (Figure 7). Taken together, our results show that LGW directly binds to the *GW7* promoter and regulates its expression to control rice grain size.

## Discussion

### Nuclear localization of low grain weight is crucial for grain size regulation

In this study, we report a rice mutant with *lgw*, which was isolated from *japonica* cv. Wuyunjing7 (WYJ7) using the heavy ion beam treatment. Map-based cloning revealed that *lgw* is a novel allele in *BC12*. Unlike the *lgw* allele, which only exhibits dwarfism and small grain size phenotypes (Figure 1 and Supplementary Figure 5), the other alleles, *bc12* and *gdd1*, not only showed reduced plant height and small grain size phenotypes but also affected cellulose microfibril deposition and wall composition, leading to the brittleness of culms phenotype (Zhang et al., 2010; Li et al., 2011). Therefore, this allele may provide a new understanding of how LGW regulates rice grain size. Different mutations in the same gene may have different biological functions. Protein alignment analysis revealed that the mutation site of *lgw* occurred in the NLS of LGW (Figure 3D), which led lgw to not localize to the nucleus (Figure 4B), which in turn may affect its function. In *Arabidopsis*, FRA1, a homolog of LGW/BC12, affects cellulose microfibril-oriented deposition in the secondary cell walls of fibers (Zhang et al., 2010). Unlike FRA1, which localizes only in the cytoplasm, LGW/BC12/GDD1 is localized in both the cytoplasm and the nucleus. The cytoplasm function of BC12 is essential for cellulose microfibril deposition and cell wall composition (Zhang et al., 2010). Given that the *lgw* mutant exhibits dwarfism and a small grain size without brittle culm phenotypes, the function of LGW/BC12 in the nucleus is crucial for plant growth and grain development. Overexpression of *LGW* led to increased grain length and weight (Figure 5), indicating that LGW is a positive regulator of grain size. Taken together with the GDD1/BC12-related research, we speculate that LGW/GDD1/BC12 is involved in secondary cell wall formation, plant growth, and grain development depending on different pathways. BC12 is involved in cellulose microfibril deposition, and the cell wall composition for brittleness depends on its functions in the cytoplasm (Zhang et al., 2010). LGW/GDD1/BC12 regulates plant growth, and grain size depends on its function in the nucleus by regulating the expression of related genes.

### Low grain weight regulates grain size through modulating *GW7* expression

LGW is the closest homolog of FRA1 (Supplementary Figure 3), which has a conserved KIF4 domain and is highly similar to other known kinesin-4 subfamily members. In addition, LGW shares the conserved domain of bZIP transcription factors that interact with CCA(N)_*n*_TGG (Li et al., 2011). These results suggest that LGW, located in the nucleus, may function as a transcription factor. *GW7*, a key QTL for regulating grain size and its expression, is regulated by the SBP-domain transcription factors GW8/OsSPL16 (Wang et al., 2015). The expression analysis revealed that the expression levels of *GW7* were significantly downregulated in *lgw* plants and upregulated in *LGW*-OE2 plants, indicating that *GW7* may be the target gene of LGW. The upstream 3,000 bp region of the *GW7* promoter has seven candidate binding elements (Supplementary Table 1), and the ChIP assay results show that LGW could bind to two of them upstream near the 1,600 bp region (Figure 6B). Furthermore, Y1H assay results demonstrate that LGW could directly bind to the promoter of *GW7* (Figure 6D). GDD1/BC12 not only has the ability to bind to the *KO2* promoter but also transactivates KO2 transcription. Transactivation analysis indicates that LGW functioned as a transcriptional activator to initiate the transcription of *GW7* (Figure 6F). Elevating *GW7* expression levels in *lgw* plants rescued the small grain size phenotype (Figure 7) and further genetically verifies LGW as a transcriptional activator that directly regulates *GW7* expression control grain size.

Therefore, our findings reveal an important genetic and molecular mechanism for grain size control involving the LGW/BC12-*GW7* regulatory module in rice, suggesting that this module is a promising target for grain size improvement in crops. Furthermore, our findings provide new insights into LGW/BC12 function in cell wall formation, plant growth, and grain size depending on its subcellular localization.

## Data availability statement

The original contributions presented in this study are included in the article/Supplementary material, further inquiries can be directed to the corresponding authors.

## Author contributions

YY, KW, XF, YW, and BL together designed the experiments. YY wrote the manuscript. YY, SW, YR, and HY performed most of the experiments. SW performed the transactivation analysis. YR performed the mutant screening. HY performed the map-based cloning. JG performed the construction of the vectors. HJ performed the ChIP assay. XZ performed the subcellular localization of LGW. WL performed the Y1H assay. YZ performed the expression pattern analysis. LT performed the field experiment. All authors have discussed the results and contributed to the drafting of the manuscript.

## Supplementary material

Supplementary Figure 1 Plant height containing each internode length. Error bars represent SE (*n* = 30).

Supplementary Figure 2 Plant height analysis of rice plants to show the rescued plant height in the complemented plants. **(A)** Morphology of mature WT, *lgw*, and complementation plants. Scale bar = 10 cm. **(B)** Plant height of WT, *lgw*, and complementation plants. Different letters denote significant differences (*P* < 0.05) from Duncan’s multiple range test.

Supplementary Figure 3 Phylogenetic tree of LGW and other kinesin-like proteins in the KIF4 subfamily. The numbers at each node represent the bootstrap support (percentage), and the scale bar is an indicator of genetic distance based on branch length.

Supplementary Figure 4 The subcellular localization of LGW and lgw. LGW-GFP and lgw-GFP were expressed in *N. benthamiana* leaves, bars = 30 μm.

Supplementary Figure 5 Comparison of the mechanical strength of WT, *cef1*, and *lgw*. The *lgw* shows the normal mechanical strength to its WT without brittle culm phenotype. The *cef1* is a typical brittle culm mutant as previously reported.

Supplementary Table 2 Primers were used in this study.
