# Supplementary material for: Low grain weight, a new allele of BRITTLE CULM12, affects grain size through regulating GW7 expression in rice
Source: Front Plant Sci. 2022 Sep 13;13:997624. doi: 10.3389/fpls.2022.997624 (PMC9513473; doi:10.3389/fpls.2022.997624)
Supplement: Supplementary file 1 [file Table_1.DOCX]

| Position | CCA(N)_n_TGG |
| --- | --- |
| -2654 | CCATTGG |
| -2247 | CCAATGG |
| -1680, -1240 | CCATTTGG |
| -1641 | CCATCATGG |
| -2943 | CCATTGTTGG |
| -1914 | CCAACGTACTGG |

Table S1 Binding elements CCA(N)_n_TGG analysis in *GW7* promoter
