# Supplementary figures and images for: Low grain weight, a new allele of BRITTLE CULM12, affects grain size through regulating GW7 expression in rice
Source: Front Plant Sci. 2022 Sep 13;13:997624. doi: 10.3389/fpls.2022.997624 (PMC9513473; doi:10.3389/fpls.2022.997624)

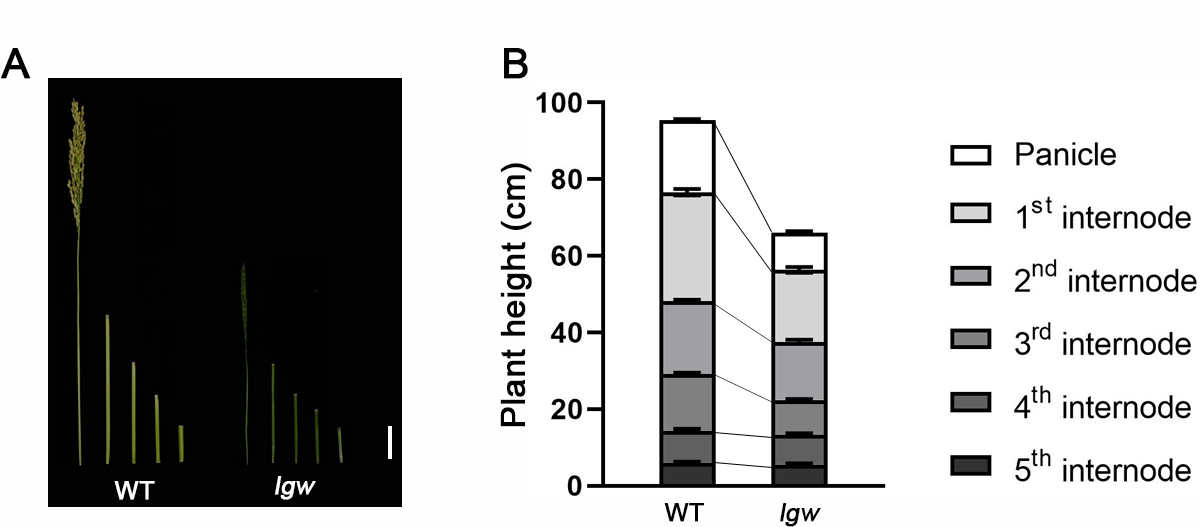

Supplement: Supplementary Figure 1 — Plant height containing each internode length. Error bars represent SE (n = 30). [file Image_1.TIF]

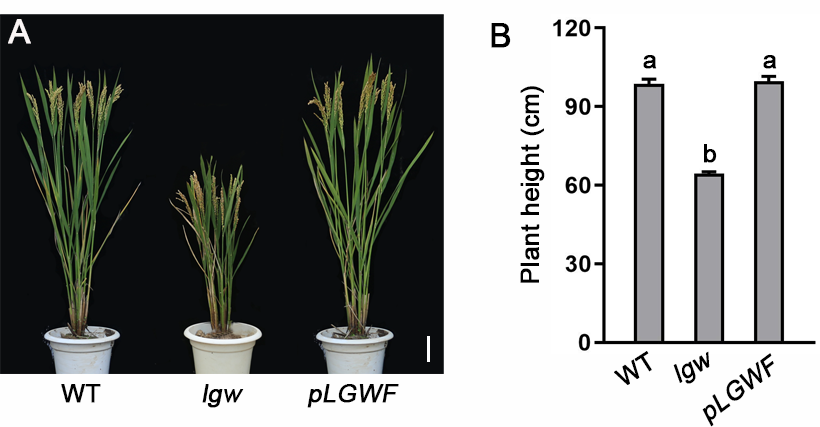

Supplement: Supplementary Figure 2 — Plant height analysis of rice plants to show the rescued plant height in the complemented plants. (A) Morphology of mature WT, lgw, and complementation plants. Scale bar = 10 cm. (B) Plant height of WT, lgw, and complementation plants. Different letters denote significant differences (P < 0.05) from Duncan’s multiple range test. [file Image_2.TIF]

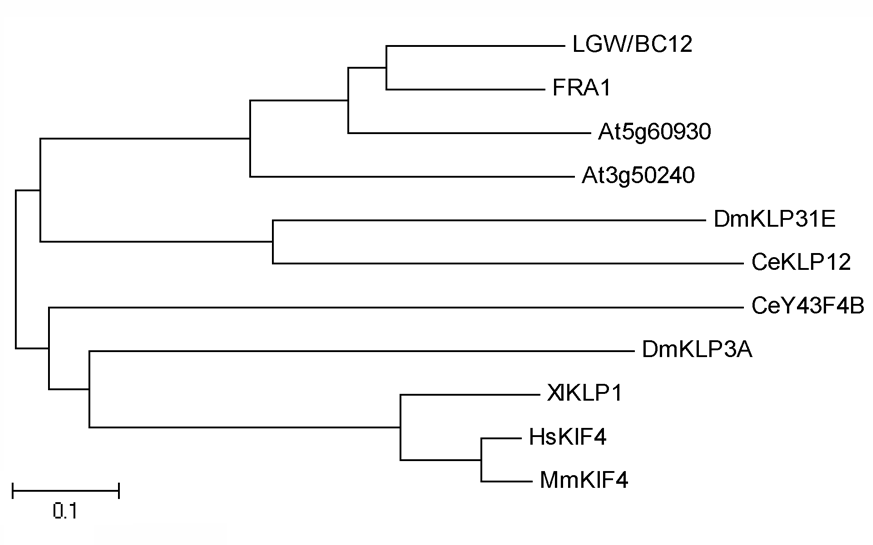

Supplement: Supplementary Figure 3 — Phylogenetic tree of LGW and other kinesin-like proteins in the KIF4 subfamily. The numbers at each node represent the bootstrap support (percentage), and the scale bar is an indicator of genetic distance based on branch length. [file Image_3.TIF]

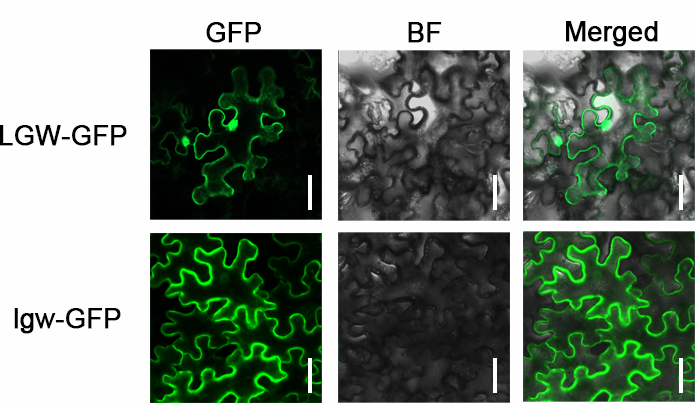

Supplement: Supplementary Figure 4 — The subcellular localization of LGW and lgw. LGW-GFP and lgw-GFP were expressed in N. benthamiana leaves, bars = 30 μm. [file Image_4.TIF]

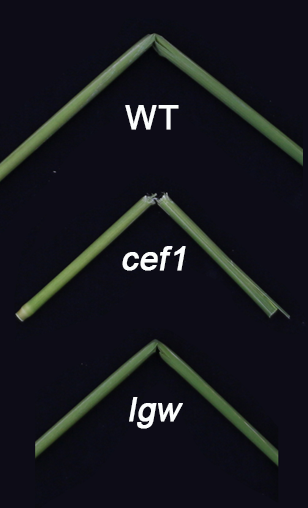

Supplement: Supplementary Figure 5 — Comparison of the mechanical strength of WT, cef1, and lgw. The lgw shows the normal mechanical strength to its WT without brittle culm phenotype. The cef1 is a typical brittle culm mutant as previously reported. [file Image_5.TIF]
